# Evidence of new intragenic *HBB* haplotypes model for the prediction of beta-thalassemia in the Malaysian population

**DOI:** 10.1038/s41598-021-96018-y

**Published:** 2021-08-18

**Authors:** Nur-Aisyah Aziz, Wan-Rohani Wan Taib, Nur-Khairunnisa Kharolazaman, Imilia Ismail, Hamid Ali Nagi Al-Jamal, Nadiah Wan-Arfah Wan Abdul Jamil, Ezalia Esa, Hishamshah Ibrahim

**Affiliations:** 1 School of Biomedicine, Faculty of Health Sciences, Universiti Sultan Zainal Abidin, Gong Badak Campus, Terengganu, Malaysia; 2 Molecular Genetics Laboratory, Haematology Unit, Cancer Research Centre, Institute for Medical Research (IMR), National Institute of Health (NIH), Ministry of Health Malaysia, Putrajaya, Malaysia; 3 Malaysian Thalassemia Registry, Medical Development Division, Ministry of Health (MOH), Putrajaya, Malaysia

**Keywords:** Biological techniques, Genetics, Molecular biology, Diseases, Medical research, Molecular medicine, Risk factors

## Abstract

This study sought to determine the potential role of *HBB* haplotypes to predict beta-thalassemia in the Malaysian population. A total of 543 archived samples were selected for this study. Five tagging SNPs in the beta-globin gene (*HBB;* NG_000007.3) were analyzed for SNP-based and haplotype association using SHEsis online software. Single-SNP-based association analysis showed three SNPs have a statistically significant association with beta-thalassemia. When Bonferroni correction was applied, four SNPs were found statistically significant with beta-thalassemia; IVS2-74T>G (*p*^*adj*^ = 0.047), IVS2-16G>C (*p*^*adj*^ = 0.017), IVS2-666C>T (*p*^*adj*^ = 0.017) and 3’UTR + 314G>A (*p*^*adj*^ = 0.002). However, 3'UTR + 233G>C did not yield a significant association with *p*^*adj*^ value = 0.076. Further investigation using combined five SNPs for haplotype association analysis revealed three susceptible haplotypes with significant *p* values of which, haplotypes 1-2-2-1-1 (*p* = 6.49 × 10^−7^, OR = 10.371 [3.345–32.148]), 1-2-1-1-1 (*p* = 0.009, OR = 1.423 [1.095–1.850] and 1-1-1-1-1 (*p* = 1.39 × 10^−4^, OR = 10.221 [2.345–44.555]). Three haplotypes showed protective effect with significant *p* value of which, 2-2-1-1-1 (*p* = 0.006, OR = 0.668 [0.500–0.893]), 1-1-2-2-1 (*p* = 0.013, OR = 0.357 [0.153–0.830]) and 1-1-2-1-1 (*p* = 0.033, OR = 0.745 [0.567–0.977]). This study has identified the potential use of intragenic polymorphic markers in the *HBB* gene, which were significantly associated with beta-thalassemia. Combining these five SNPs defined a new haplotype model for beta-thalassemia and further evaluation for predicting severity in beta-thalassemia.

## Introduction

Haemoglobin disorders are the most common monogenic disease worldwide^1,2^. These inherited autosomal recessive disorders are classified according to the haemoglobin expression or synthesis. There are three main categories of haemoglobin disorders; haemoglobinopathy is the structurally abnormal haemoglobin, thalassemia is the quantitatively reduced haemoglobin^3,4^ and, hereditary persistence of foetal haemoglobin (HPFH) and δβ-thalassemia are characterized by increased levels of foetal haemoglobin (HbF) in adulthood. The prevalence of haemoglobin disorders was high in tropical and subtropical regions such as sub-Saharan Africa, Mediterranean, Middle East, Indian subcontinent, and Southeast Asia^3^. However, due to modernization, people from epidemic areas migrated to the non-epidemic area. Hence, haemoglobin disorders have become a significant health problem in 71% of 229 countries globally^5^.

The recent report by the Malaysian Thalassemia Registry (MTR) has recorded a total of 8681 thalassemia cases from 2007 until November 2018. Since the launch of the National Thalassemia Preventive and Control Program in 2004, healthcare facilities have been upgraded to provide better quality for patient management. Hence, the survival rate of patients with thalassemia in Malaysia has improved^6^. With several molecular studies have been done previously in the East and West Malaysia, genetic heterogeneity is more observed in multiracial population in Malaysia with a diverse spectrum of alpha (α-), beta (β-) and delta (δ-) globin genes mutations among the patients with thalassemia syndromes^7–11^. Beta-thalassemia is due to decreased beta-globin chain synthesis of which, caused by a mutation in the *HBB* gene. The *HBB* gene mapped on chromosome 11p15.4 with a region spanning from 5,225,464 to 5,229,395 bp on the reverse strand^12^. Therefore, the identification of nucleic acid variations in the *HBB* gene has improved our understanding of underlying causal mutations of beta-thalassemia in Malaysia.

The clinical presentation and molecular circumstantial of beta-thalassemia are highly heterogeneous and dependent on geographical and ethnic factors^13^. The evidence may be derived from genetic predisposition, which is unique to particular ethnic groups, and that could enable targeted molecular analysis being designed^14,15^. Genetic heterogeneity is more observed among different ethnicity in Malaysia with a diverse spectrum of *HBB* gene mutations. According to Elizabeth &Ann (2010), approximately 73% of Malay patients with beta-thalassemia were due to mutation at codon 26(A>G) or HbE (β^E^), IVS1-5(G>C) (severe β^+^) and IVS1-1(G>T) (β°). Whilst for Malaysian Chinese, 90% of beta-thalassemia cases were due to codon 41/42 (-TTCT) (β°), IVS2-654(C>T), -28(A>G), codon 17(A>T) and codon 71/72(+ A). In East Malaysia, especially in Sabah, 90% of the Kadazan-Dusun with beta-thalassemia were due to β-Filipino deletion (β°)^14,15^.

However, the genetic variant interaction in conferring the effect based on haplotype inference has yet to be explored and refined in beta-thalassemia among the Malaysian population. Deciphering the predisposing effect by the potential haplotype markers can promote the exposition of underlying mechanisms of thalassemia development. In this study, five single nucleotide polymorphisms (SNPs) within the *HBB* gene were evaluated to determine its significance and haplotype structure inference with beta-thalassemia in Malaysia, which was the first study conducted in Malaysia to the best of our knowledge.

## Results

### Single association analysis

In single-based association analysis, three tagging SNPs at IVS2-16G>C, IVS2-666C>T and 3’UTR + 314G>A showed a statistically significant association with beta-thalassemia with *p* value of 0.036, OR = 1.300 [1.017–1.66]; 0.032, OR = 0.765 [0.598–0.978] and 0.004, OR = 2.013 [1.238–3.272], respectively. However, tagging SNP at IVS2-74T>G and 3'UTR + 233G>C did not show statistically significant association with beta-thalassemia of which, the *p* value of 0.099 and 0.211. The minor allele of these two variants showed a trend towards protective effect based on odds ratios less than 1 (OR = 0.794 [0.604–1.044] and OR = 0.663 [0.347–1.267] respectively).

Table 1 depicts the genotypic association analysis of the five assigned SNPs. The most common genotype for IVS2-74T>G, IVS2-16G>C, IVS2-666C>T, 3’UTR + 233G>C and 3’UTR + 314G>A was TT (59.8% in case and 52.6% in control group), GC (49% in case and 49.1% in control group), CT (43% in case and 48.5% in control group), GG (94.4% in case and 91.5% in control group) and GG (82.3% in case and 90.4% in control group) respectively. Three tagging SNPs (IVS2-74T>G, 3'UTR + 233G>C, and 3'UTR + 314G>A) showed a high homozygosity rate in case and control groups. Meanwhile, a high heterozygosity rate was found in IVS2-16G>C and IVS2-666C>T in both groups.

**Table 1:** Single association analysis of five tagging SNPs of the *HBB* gene with beta-thalassemia.

SNP	Genotype data (frequency)				Case–control analysis			
	1/1	1/2	2/2	HWE (*p* value)	MAF	*P* value	OR [95%CI]	*p*^*adj*^ value
IVS2-74T>G	**149 (0.598)**	81 (0.325)	19 (0.076)	0.096	119 (0.239)	0.099	0.794 [0.604–1.044]	0.047
	**154 (0.526)**	112 (0.382)	27 (0.092)	0.316	166 (0.283)			
IVS2-16G>C	99 (0.398)	**122 (0.490)**	28 (0.112)	0.293	178 (0.357)	0.036	1.300 [1.017–1.66]	0.017
	98 (0.334)	**144 (0.491)**	51 (0.174)	0.879	246 (0.420)			
IVS2-666C>T	106 (0.426)	**107 (0.430)**	36 (0.145)	0.292	179 (0.359)	0.032	0.765 [0.598–0.978]	0.017
	98 (0.334)	**142 (0.485)**	53 (0.181)	0.900	248 (0.423)			
3'UTR + 233G>C	**237 (0.944)**	13 (0.052)	1 (0.004)	0.091	15 (0.030)	0.211	0.663 [0.347–1.267]	0.076
	**268 (0.915)**	24 (0.082)	1 (0.003)	0.600	26 (0.044)			
3'UTR + 314G>A	**205 (0.823)**	42 (0.169)	2 (0.008)	0.925	46 (0.092	0.004	2.013 [1.238–3.272]	0.002
	**263 (0.904)**	28 (0.096)	0 (0.000)	0.389	28 (0.048)			

Case data is at the top line, while control data is at the bottom line. The major allele is depicted as 1. Minor allele is represented as 2. *MAF* minor allele frequency. The *p* value < 0.05 is considered significant in Pearson Chi-Square. *p*^*adj*^-value is Bonferroni correction based on the five markers tested in this study.

All these SNPs were also tested for pairwise linkage disequilibrium (LD) across the *HBB* gene by the square of the correlation coefficient (r^2^). No deviation in Hardy–Weinberg equilibrium (HWE) for all five SNPs in the control and case group (0 < *p* < 1). After the Bonferroni correction, four SNPs were found significantly associated with beta-thalassemia; IVS2-74T>G (*p*^*adj*^ = 0.047), IVS2-16G>C (*p*^*adj*^ = 0.017), IVS2-666C>T (*p*^*adj*^ = 0.017) and 3’UTR + 314G>A (*p*^*adj*^ = 0.002).

### Haplotype analysis

Captivated by this favorable data in single association analysis, further investigation was conducted using combined allele from IVS2-74T>G, IVS2-16G>C, IVS2-666C>T, 3'UTR + 233G>C and 3'UTRt + 314G>A of *HBB* gene in an attempt to evaluate the predisposing effect of *HBB* intragenic haplotype with beta-thalassemia. The naming system for the haplotype in this study is not related to the system used in the previous PCR–RFLP based haplotyping studies. Haplotype analysis revealed significant association for three haplotypes; 1-2-2-1-1, 1-2-1-1-1 and 1-1-1-1-1 with susceptibility effect towards beta-thalassemia of which, the *p* values were 6.49 × 10^−7^ (OR = 10.371 [3.345–32.148]), 0.009 (OR = 1.423 [1.095–1.850] and 1.39 × 10^−4^ (OR = 10.221 [2.345–44.555]) respectively. On the other hand, three haplotypes; 2-2-1-1-1, 1-1-2-2-1 and 1-1-2-1-1 significantly conferred an opposing manner of effect to beta-thalassemia with the *p* value 0.006 (OR = 0.668 [0.500–0.893]), 0.013 (OR = 0.357 [0.153–0.830]) and 0.033 (OR = 0.745 [0.567–0.977]) respectively. However, one haplotype with allele combinations 1-1-2-1-2 did not show any significant association with beta-thalassemia, where the *p* value was 0.899, yet the trend was towards susceptibility as depicted by OR = 1.041 [0.559–1.939]). The summary of these findings was tabulated in Table 2. Haplotype with the frequency < 0.03 in both controls and cases were automatically excluded from the analysis by the SHEsis online software.

**Table 2:** Haplotype analysis of IVS2-74T>G, IVS2-16G>C, IVS2-666C>T, 3'UTR + 233G>C and 3'UTR + 314G>A in all races dataset with 249 cases and 294 controls.

**Haplotype	*Case (frequency)	*Control (frequency)	*p* value	OR [95% CI]
2-2-1-1-1	95.08 (0.192)	164.67 (0.284)	0.006	0.668 [0.500-0.893]
1-2-2-1-1	26.38 (0.053)	3.43 (0.006)	6.49 × 10^−7^	10.371 [3.345–32.148]
1-2-1-1-1	167.60 (0.338)	169.25 (0.292)	0.009	1.423 [1.095–1.850]
1-1-2-2-1	7.08 (0.014)	24.70 (0.043)	0.013	0.357 [0.153–0.830]
1-1-2-1-2	18.79 (0.038)	23.11 (0.040)	0.899	1.041 [0.559–1.939]
1-1-2-1-1	120.08 (0.242)	188.91 (0.326)	0.033	0.745 [0.567–0.977]
1-1-1-1-1	15.61 (0.031)	2.02 (0.003)	1.39 × 10^−4^	10.221 [2.345–44.555]

*Frequency < 0.03 in both controls and cases has been dropped in the analysis.

**Major allele is depicted as 1. **Minor** allele is depicted as 2. A sequential in allele combination represents for IVS2-74T>G, IVS2-16G>C, IVS2-666C>T, 3'UTR + 233G>C and 3'UTR + 314G>A respectively.

## Discussion

In this study, we explored a single-based and haplotype association of five intragenic *HBB* polymorphisms in beta-thalassemia cases from Malaysia. It was suggested that the association of intragenic SNPs might be useful for the diagnosis and delineation of the clinical heterogeneity of beta-thalassemia^16^. Furthermore, the intragenic SNPs could be useful marker for linkage analysis and in prenatal diagnosis it can improve the diagnostic errors of which, caused by recombination^17^.

From the analysis of single-based association, two intronic polymorphisms; IVS2-16G>C, and IVS2-666C>T, and one variant at 3' untranslated region to *HBB* gene assigned as 3'UTR + 314G>A were found significantly associated with beta-thalassemia. The substitution of C to T allele at position 666 of intron 2 with minor allele frequency (MAF) of 0.359 in case group and 0.423 in control group conferring protection in beta-thalassemia with the odds ratio of 0.765 (*p* = 0.032). However, we noted that the MAF for IVS2-666C>T from this study was higher when compared with global MAF in the ClinVar (0.286) database but lower compared to 1000 Genome Project (0.713)^18^. The genotype distribution of this intronic polymorphism revealed the heterozygote had yielded the highest frequency (48.5%) in the control group. Association study done by Akhavan-Niaki et al. (2011) reported that IVS2-666C>T was found to be linked to a mutation at codon 8(-AA) [*HBB:c.*25_26delAA], of which this β°-mutation was mainly described among the population from the Middle East and the Mediterranean. Hence, the authors suggested that IVS2-666C>T would be useful as a marker for codon 8 genotyping in prenatal diagnosis^17^.

Meanwhile, two other variants showed a significant susceptibility effect towards beta-thalassemia: IVS2-16G>C and 3'UTR + 314G>A. The MAF for IVS2-16G>C was 0.357 in the case group and 0.420 in the control group conferring susceptibility in beta-thalassemia with the odds ratio of 1.300 (*p* = 0.036). In comparison to global MAF from the ClinVar database (0.280), MAF findings for IVS2-16G>C in this study were noted higher but lower when compared to the 1000 Genomes Project (0.720)^19^. The untranslated region (UTR) is the sequence in the 3' region of a gene but not translated during protein synthesis and contains regulatory element for the gene expression^20^. A variant in the 3'UTR of the *HBB* gene*,* which is assigned as 3'UTR + 314 G>A was found to have a significant susceptibility effect towards beta-thalassemia with the odds ratio 2.013 (*p* = 0.004). The MAFs were found to be 0.092 in the case group and 0.048 in the control group. However, we noted that there was very limited report of this variant in the literature for further comparison. Overall, we noticed that the MAF for the three significant variants in this study were within the range of global MAF from other studies reported in the ClinVar database^18,19^. The different MAF value could be varied across diverse ethnic or population as well as study sample size^21^.

In an attempt to further evaluate the role of *HBB* haplotypes in beta-thalassemia in Malaysia, haplotype analysis revealed several susceptible and protective haplotypes^22^. The potential applications of haplotype-tagged SNPs have been widely described in the literature. Fields of application include, for example, disease association and pharmacogenetic studies^23^. Protective haplotype had been observed in association with breast density in fine mapping analysis^24^. In this study, we identified seven different haplotypes using the five intragenic *HBB* SNPs. A comparable finding was reported by Bilgen et al. (2011) for the haplotype analysis in the Turkish population. Likewise, the authors have also reported that SNP based haplotyping using five intragenic SNPs has successfully established the beta globin gene mutation related haplotypes^16^. In the earlier studies done by Fuchareon et al. (2001) and Sanguansermri et al. (2004) also have reported association of certain haplotype pattern with HbE and common beta-thalassemia mutation respectively by using PCR–RFLP method. To the best of our knowledge, no study was done so far to evaluate the important role of intragenic *HBB* SNPs in thalassemia syndrome in Southeast Asian region. In this study, we identified six significant haplotypes of which, have important role for beta-thalassemia. Noteworthy, individuals with haplotype that consists of all major alleles from our assigned *HBB* polymorphisms (1-1-1-1-1) might have a higher risk in developing beta-thalassemia. However, if the minor allele from IVS2-666C>T is substituted, the effect becomes a protective effect. This allele transition might reveal the protective role from the minor allele of IVS2-666C>T. The same effect is reflected in IVS2-16G>C. However, the protective effect from the minor allele of IVS2-16G>C was not strong enough to confer susceptibility for this haplotype.

Interesting to note that the combination of both minor alleles from IVS2-666C>T and 3'UTR + 233G>C with other dominant alleles projected higher protection, which elucidates the same protective role from 3'UTR + 233G>C. These synergist effects provide a better outcome for individuals with this haplotype 1-1-2-2-1. The same synergist effect was also observed for haplotype 2-2-1-1-1, which revealed the protective role from IVS2-74T>G and IVS2-16G>C. Likewise, the allele substitution for 3'UTR + 314G>A in haplotype 1-1-2-1-2 dropped the protective effect from haplotype 1-1-2-1-1. The susceptible effect might explain this from a minor allele of 3'UTR + 314G>A. This haplotype-based association analysis was carried out to provide a prediction of the predisposing effect and reveal the severity and possible prognosis using haplotype-tagged SNPs of *HBB* gene for beta-thalassemia. Thus, this model could be further developed for the improvement of clinical management of beta-thalassemia in Malaysia mainly based on the personalized haplotype profile.

In conclusion, the presented study the first study on intragenic polymorphic markers of the beta-globin gene involving the Malaysian population. Identification of susceptible and protective haplotype markers that conferred the significant association with beta-thalassemia in Malaysia can be further refined following the multi-ethnic background of the Malaysian population. The association data on a single genotype and haplotype might disclose the effect of *HBB* polymorphisms in beta-thalassemia that might provide an impact in the understanding of beta-thalassemia propensity. This study can be ascertained by larger sample size, and stratification by ethnicity should be deliberated since Malaysia is inhibited by various ethnicity.

## Materials and methods

### Study population

This cross-sectional study was conducted among the referral case for DNA analysis of thalassemia syndromes in the Institute for Medical Research (IMR), Kuala Lumpur. The study protocol was approved by the Medical Research Ethics Committee [MREC; NMRR-18-3977-43849 (IIR)] and UniSZA Human Research Ethical Committee [UniSZA/UHREC/2020/170]. Informed consent was obtained from each case prior blood collection was done. Protocol of this study was in accordance with the Declaration of Helsinki. A total of 543 (294 controls & 249 cases) archived cases from the year 2011 until 2014 were reviewed for this study. Only cases with valid Malaysian identity card numbers were included in this study. Cases with no sequencing results and no valid Malaysian identity card numbers were excluded from this study. These cases were molecularly ascertained via Sanger sequencing using 3730XL DNA Analyser (Applied Biosystem, Foster City, CA, USA) for the presence of *HBB* gene variation. Samples with heterozygous or compound heterozygous or homozygous state of *HBB* gene mutations were grouped as cases. Whilst controls were the samples without the known beta-globin gene mutation.

### SNP genotyping

Genomic DNA was extracted from peripheral blood using a commercial DNA extraction kit (QIAGEN, Germany). The detection of the genotype for IVS2-74T>G (HBB:c.315 + 74T>G), IVS2-16G>C (HBB:c.315 + 16G>C), IVS2-666C>T (HBB:c.316-185C>T), 3'UTR + 233G>C (HBB:c.*233G>C) and 3'UTR + 314G>A (HBB:c.*314G>A) polymorphic site in the *HBB* gene was performed using a direct DNA sequencing technique in which the cycle sequencing used the BigDye® Terminator v3.1 cycle sequencing kit. Sequence analysis was performed on CLC Main Workbench 6 version 6.6.1 software (CLC Bio, Denmark).

### Bioinformatics analysis

The SHEsis Online software (http://analysis.bio-x.cn/myAnalysis.php) was employed to assess the Hardy–Weinberg equilibrium, allele frequency, SNPs and haplotype association in which allelic and genotypic distribution were compared between case and control groups^25^. Bonferroni correction was used for multiple comparison correction. The odds ratios (ORs) value with a 95% confidence interval (95% CI) in which a *p* value of 0.05 was considered as significant.
